# Comparison between second generation HydroSoft coils and bare platinum coils for the treatment of large intracranial aneurysms

**DOI:** 10.1177/15910199221088711

**Published:** 2022-03-23

**Authors:** Agostino Tessitore, Aldo Paolucci, Sophia Hohenstatt, Antonio A Caragliano, Orazio Buonomo, Enricomaria Mormina, Antonio Pitrone, Sergio L Vinci

**Affiliations:** 1 Neuroradiology Unit, University Hospital A.O.U. Policlinico “G. Martino” - Messina, Italy; 2 Operative Unit of Neuroradiology, 9339 Fondazione IRCCS Ca’ Granda Ospedale Maggiore Policlinico di Milano, Milan, Italy; 3 Department of Neuroradiology, 27178 Heidelberg University Hospital, Heidelberg, Germany; 4 Neuroradiology Unit, University Hospital A.O.U. “G. Martino” Messina, Italy; 5 Department of Biomedical, Dental, Morphological and Functional Imaging Sciences, 18980 University of Messina, Italy

**Keywords:** Large aneurysms, Hydrocoils, cerebral aneurysm

## Abstract

**Background:**

The development of HydroSoft coils (HSC) aims to reduce the high recurrence and retreatment rates observed in large brain aneurysms by improving primary brain aneurysm filling and thus occlusion efficacy. We compared clinical and angiographic effectiveness of bare platinum coils (BPC) versus second generation HSC for large intracranial aneurysms at our center.

**Methods:**

We included 61 large aneurysms between 2015 and 2018, 29 embolized primarily using HSC and 32 treated with BPC. The aneurysm occlusion rates were assessed after 3 and 12 months with an MRI scan and at 6 moths with a control digital subtraction angiography (DSA) using the Raymond-Roy occlusion classification (RROC). Clinical outcomes were evaluated using the modified ranking scale (mRS).

**Results:**

The observed immediate occlusion rate was slightly better in the BPC group, however, this group had a significant increase of progressive reperfusion at all imaging follow-up. Contrarily, the rate of complete occlusion increased significantly in the HSC group, starting from the 6 and 12-months follow-up. 7 aneurysms (11.4%) were re-treated (15.6% BPC and 6.9% HSC). The 6 and 12-months clinical data showed mRS score 0-1 in 96.7% of patients.

**Conclusions:**

In our single-center experience, the second generation HydroSoft coils were shown to be safe and effective for endovascular treatment of large intracranial aneurysms with encouraging clinical and angiographic results, also for ruptured aneurysms. Even if the validity is limited due to our small cohort size, HSC showed a significantly lower rate of recurrence at mid-term follow-up when compared to BPC.

## Introduction

Since the introduction of helical platinum coils in the early 1990s,^1^ many technical developments were proposed to increase aneurysm occlusion rates to improve long-term outcome and reduce recurrence. The Hydrocoil Embolic System (MicroVention Inc.) is a new technological device developed to increase the effectiveness of intrasaccular embolization. It consists of porous hydrogel that either surrounds the platinum coil as in the 1^st^ generation or is surrounded by the platinum coil as in the 2^nd^ generation, the last one being developed for improving the handling properties.^2^ Once exposed to the biological pH 3.2 the cross-linked hydrophilic copolymers swell ameliorating the primary aneurysm filling. Our aim was to evaluate the safety and efficacy of HydroSoft coils (2^nd^ generation HSC) in the treatment of large and giant cerebral aneurysms at our premises. These types of aneurysms are particularly prone to recurrence, re-growth, and rupture.^3^ A direct comparison between HSC and bare platinum coils (BPC) was therefore performed to assess any differences in terms of outcome, recurrence rates and complications.

## Materials and methods

### Patient cohort

We performed a retrospective review of all large and giant cerebral aneurysms at our institute from 2015 to 2018 considering the ones that were treated with intrasaccular embolization therapy either with or without stent-assisted coiling (SAC or NAC, respectively). This cohort included both ruptured and unruptured aneurysms.

### Antiplatelet therapy

All patients with unruptured aneurysms underwent dual antiplatelet therapy (DAP) 7-10 days before the endovascular procedure (aspirin 100 mg and clopidogrel 75 mg); when a stent had been deployed, DAP was continued for additionally 3–6 months. Then mono-antiplatelet therapy was administered at least until the 1-year follow-up. If the deployment of the stent was necessary in patients who presented acute ruptured aneurysms, these received a loading-dose of oral clopidogrel 300 mg and 500 mg i.v. aspirin or a full i.v. dose of a glycoprotein IIb-IIIa inhibitor (Abciximab).

### Endovascular procedures

**Figure 1.**
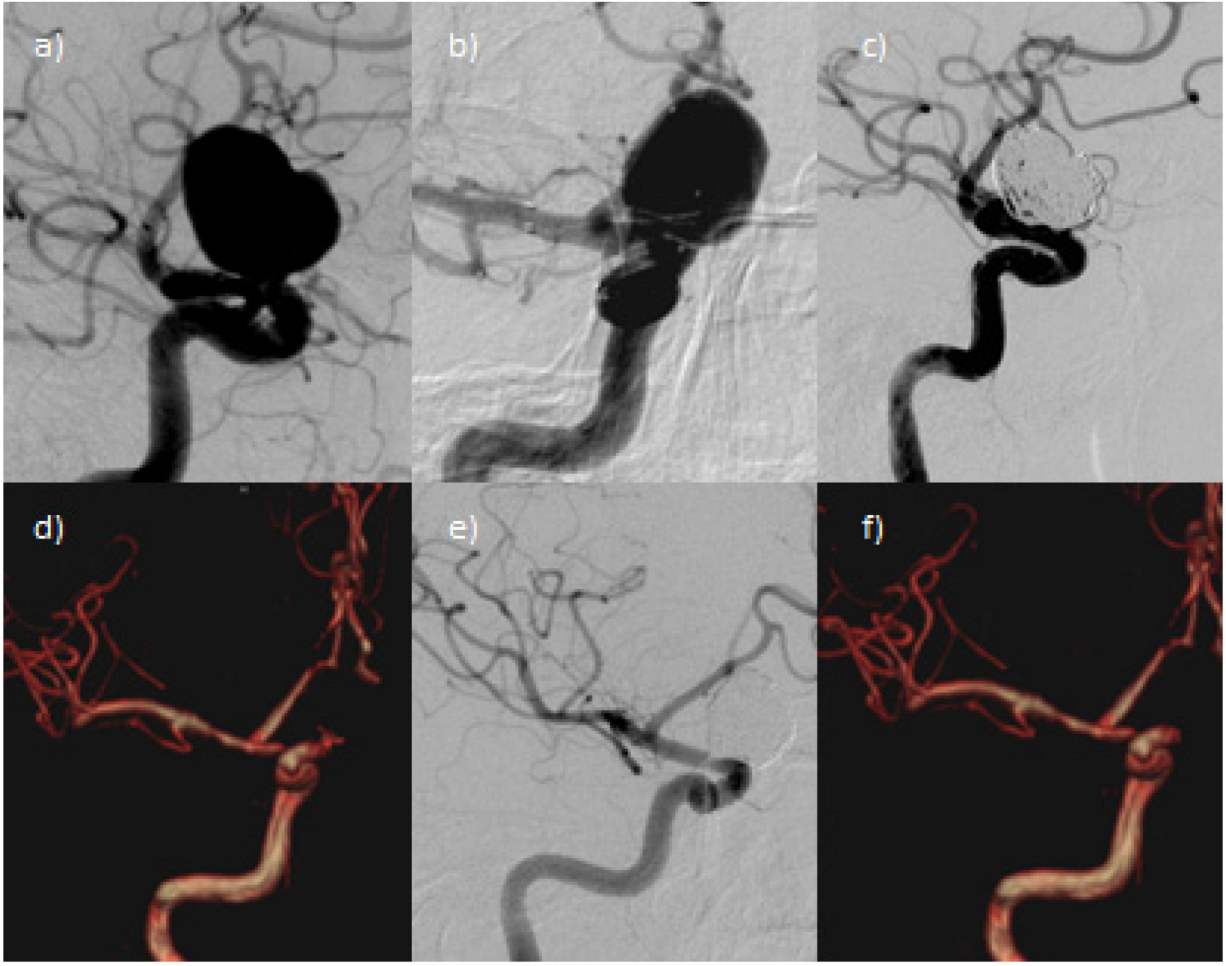
Unruptured supraclinoid ICA aneurysm treated by SAC with neuroform atlas stent and hydroSoft coils. a) DSA shows the aneurysm at the supraclinoid segment of right internal carotid artery (ICA) (max. width 18 mm); b) Angiographic vision of the Neuroform Atlas stent deployed between the M1-segment of the middle cerebral artery (MCA) and homolateral ICA, covering the aneurysmal neck. A microcatheter is positioned in the aneurysmal sac. c) The final angiogram shows a minimal neck remnant (RROC 2). d) The 3-months follow-up MRI confirms the RROC 2. e) The 6-months DSA control demonstrates complete occlusion of the aneurysmal sac (RROC 1); f) The 12-months MRI follow-up confirms RROC 1.

All neuro-endovascular treatments were performed under general anaesthesia. The femoral artery was accessed through a 6F or 8F short sheath introducer. A 6F guide catheter (Envoy, Cordis, Bridgewater, New Jersey, USA) or a 6F long sheath (Neuron Max 088 Penumbra, USA) was placed proximally in the internal carotid or vertebral artery. The aneurysm was catheterized with a 0.0165” or 0.017” microcatheter (Excelsior SL-10, Stryker neurovascular, Kalamazoo, MI, USA or Echelon 10, Medtronic Irvine, CA, USA) over a 0.012” or 0.014” neurovascular micro-guidewire to deploy the coils into the aneurysmal sac. In the case of SAC, the parent vessel was first catheterized with a suitable microcatheter over a 0.014” neurovascular micro-guidewire to deploy the stent across the aneurysmal neck. Both braided (BR) and laser-cut stents (LC) were used. Final angiograms were performed to evaluate the patency of the parent artery and to assess the aneurysm occlusion rate by using the Raymond-Roy occlusion classification (RROC).^4^ Figure 1 shows a representative case illustrating the procedure.

### Radiologic and clinical follow-up

Immediate and mid-term clinical and angiographic results were assessed. The radiological follow-up protocol included an MRI scan after 3 and 12 months and a digital subtraction angiography (DSA) after 6 months. Aneurysmal occlusion was estimated using the RROC. Recurrence was defined as any morphologic deterioration in the appearance of the aneurysm as well as a shift to a higher RROC class. The clinical evaluation was performed by an expert neurologist before the procedure, at discharge and at the 3 and 12-month follow-up. The functional outcome was assessed by the modified ranking scale (mRS), assuming the 0–1 score as a good clinical outcome.^5^

### Statistical analysis

Categorical variables are reported both as absolute and as percentage frequencies, numerical as mean and standard deviation. There has been the implementation of a non-parametric approach, due to the limited sample size and the non-normality of the distributions. The Wilcoxon test was applied to evaluate the existence of statistically significant differences for the rate of aneurysmal occlusion (RROC) for the two arms at different times (baseline vs. 3 months, baseline vs. 6 months, etc.). The Kruskal-Wallis test was additionally used to compare the average RROC values at baseline, 3, 6, and 12 months in three subgroups based on the types of stents (no stents, LC stent or BR stent) in BPC and HSC subjects. The Mann-Whitney test has been applied to evaluate the average values of RROC for all patients enrolled in the series and for the two arms of the study (BPC and HSC patients). An additional distinction has been done for no-stent, LC stent and BR stent patients. This test was also applied to proceed to pair comparison between no-stent versus LC stent, no-stent versus BR and LC stent versus BR stent. χ2 test was applied to compare the mRS values at baseline, 6, and 12 months for the two types of coils (BPC and HSC). Finally, the McNemar test was chosen to compare the baseline and 12 months mRS separately for BPC and HSC subjects. The statistical analyses were performed using statistical software SPSS ver.22 for Windows, setting the values p < 0,05 as being statistically significant.

## Results

### Population cohort and aneurysm characteristics

Out of the sixty-one patients identified for this study case (39 females; median age 64 ± 12,7 y) with 61 aneurysms, thirty-six patients (59%) had an unruptured aneurysm (17 HSC, 19 BPC). Twenty-five patients (41%) had history of acute SAH (12 HSC, 13 BPC). Concerning the aneurysms, 56 were located in the anterior circulation (27 supraclinoid segment of internal carotid artery (ICA), 15 middle cerebral artery (MCA), 14 anterior communicating artery (AcoA)). The remaining 5 were posterior circulation aneurysms, all located at the basilar artery. Table 1 summarises the main patient and aneurysm characteristics.

**Table 1.** Patients and aneurysms characteristics. Internal carotid artery (ICA); middle carotid artery (MCA); anterior communicating artery (AcoA); basilar artery (BA); subarachnoid haemorrhage (SAH); hydrosoft coils (HSC); bare platinum coils (BPC).

	All		HSC		BPC	
		SAH		SAH		SAH
Total No. of patients	61	25	29	12	32	13
Sex						
Male	22 (36)		11 (38)		11 (34.4)	
Female	39 (64)		18 (68)		21 (65.6)	
Mean age	64 ± 12,6		62,5 ± 11,2		65,4 ± 11,2	
Aneurysm characteristics						
Width	16,8 ± 4,8		17,2 ± 4,9		16,5 ± 4,8	
Height	12,1 ± 4,8		11,5 ± 5,6		12,6 ± 5,5	
Neck	4,6 ± 1,2		4,5 ± 1,1		4,7 ± 1,2	
Aneurysm site		*SAH*		*SAH*		*SAH*
ICA supraclinoid	27 (44,3)	12 (48)	15 (51,7)	7 (58,3)	12 (37,5)	5 (38,5)
MCA	15 (24,6)	5 (20)	7 (24,1)	2 (16,6)	8 (25)	3 (23)
AcoA	14 (23)	7 (28)	5 (17,2)	2 (16,6)	9 (28,1)	5 (38,5)
BA	5 (8,2)	1 (4)	2 (7)	1 (8,5)	3 (9,4)	0

Mean ± SD or number (%) is presented.

29 aneurysms were coiled either wholly or partially (>75% of the total coil length deployed) with the HSC, whereas the remaining aneurysms were coiled with BPC. SAC was used in 33 wide-necked large aneurysms (16 HSC - 1 of these being acutely ruptured, 17 BPC). Table 2 gives an overview of the type of stents used.

**Table 2.** Additional techniques performed - SAC. Stent-assisted coiling (SAC); subarachnoid haemorrhage (SAH); hydrosoft coils (HSC); bare platinum coils (BPC).

	All		HSC		BPC	
		SAH		SAH		SAH
Total No. of patients	61	25	29	12	32	13
No. of SAC – stent type	33 (54)	3 (12)	16 (55)	3 (25)	17 (53)	0
Laser-cut stent	24 (73)	1 (33.3)	12 (75)	1 (33.3)	12 (71)	0
Braided stent	9 (27)	2 (66.6)	4 (25)	2 (66.6)	5 (29)	0

Number (%) is presented.

### Technical results

A complete obliteration at the immediate post-procedural DSA was achieved in 70,5% of all cases and residual neck remnants were documented in 29,5%. Table 3 gives an overview of immediate angiographic outcomes, with the BPC group showing a slightly better immediate occlusion rate.Class 1 of RROC was obtained in 69% in the HSC group vs. 71,9% in the BPC group; Class 2 of RROC in 31% in the HSC group vs. in 28,1% in the BPC group. No Class 3 of RROC were registered at this point. In a subgroup of patients with SAH, 15 patients (60%) had an immediate complete obliteration (7 HSC, 8 BPC) and 10 (40%) showed a residual neck (5 in each group).

**Table 3.** Immediate angiographic RROC - HSC vs. BPC (SAH). Raymond–Roy occlusion classification (RROC); subarachnoid haemorrhage (SAH); hydrosoft coils (HSC); bare platinum coils (BPC).

	All		HSC		BPC	
		SAH		SAH		SAH
Complete obliteration (1)	43 (70,5)	15(60)	20 (69)	7 (58.4)	23 (71,9)	8 (61.5)
Residual neck (2)	18 (29,5)	10 (40)	9 (31)	5 (41.6)	9 (28,1)	5 (38.5)
Residual aneurysm (3)	0	0	0	0	0	0

Number (%) is presented.

### Complications

Intraoperative complications directly related to the treatment occurred in six cases (9.8%): 2 thromboembolic events in each group, one intraprocedural aneurysm rupture in the BPC group and an one HSC displacement. The displaced HSC was removed without any thromboembolic complications. Acute thromboembolic phenomena were managed by injecting a full i.v. dose of anti-glycoprotein IIb-IIIa, achieving the complete regression of the thrombosis at all instances. At discharge, no deterioration of the mRS was registered. In the study-case, we registered no cases of intra- and peri-procedural death directly related to the intervention.

### Efficacy outcome

All the patients included in our analysis completed the radiological follow-up.

At the 3-months MRI follow-up 17 (58.6%) aneurysms of the HSC group were completely occluded whereas 12 of them (41.4%) showed a residual neck. At the 6-month DSA the complete occlusion rates of the HSC group went up to 23 cases (79.3%), a residual neck being still documented in 6 cases (20.7%). Finally, at the 1-year MRI the aneurysms coiled with HSC presented a complete occlusion in 24 cases (82.8%), a residual neck in 5 cases (17.2%) with no residual aneurysms. Comparing the rate of aneurysmal occlusion in BPC group versus HSC group there were statistically significant differences in the frame of the 3, 6 and 12-months follow-ups with higher RROC values impacting the BPC group. In the 25 SAH patients, those coiled with HSC presented a higher rate of complete aneurysmal occlusion (RROC class 1) during the 6 and 12-months follow-ups. A total of 7 aneurysms were retreated at 1 year, five class 3 of RROC aneurysms of the BPC group and two class 2 of RROC of the HSC group. Table 4 summarizes the efficacy outcomes in the different patient subgroups.

**Table 4.** Efficacy outcome (RROC). Raymond–Roy occlusion classification (RROC); magnetic resonance imaging (MRI); digital subtraction angiography (DSA); subarachnoid haemorrhage (SAH); hydrosoft coils (HSC); bare platinum coils (BPC).

	All		HSC		BPC	
		(SAH)		(SAH)		(SAH)
***3-months MRI follow-up***	* *	* *	* *	* *	* *	* *
Complete obliteration (1)	29 (47.5)	9 (36)	17 (58.6)	6 (50)	12 (37.5)	3 (23.1)
Residual neck (2)	29 (47.5)	14 (56)	12 (41.4)	6 (50)	17 (53.1)	8 (61.5)
Residual aneurysm (3)	3 (5)	2 (8)	0	0	3 (9.4)	2 (15.4)
***6-months DSA follow-up***	* *		* *		* *	
Complete obliteration (1)	35 (57.4)	12 (48)	23 (79.3)	9 (75)	12 (37.5)	3 (23.1)
Residual neck (2)	23 (37.7)	12 (48)	6 (20.7)	3 (25)	17 (53.1)	9 (69.2)
Residual aneurysm (3)	3 (4.9)	1 (4)	0	0	3 (9.4)	1 (7.7)
***12-months MRI follow-up***	* *		* *		* *	
Complete obliteration (1)	35 (57.4)	11 (44)	24 (82.8)	9 (75)	11 (34.4)	2 (15.4)
Residual neck (2)	21 (34.4)	13 (52)	5 (17.2)	3 (25)	16 (50)	10 (76.9)
Residual aneurysm (3)	5 (8.2)	1 (4)	0	0	5 (15.6)	1 (7.7)

Number (%) is presented.

### Safety outcome

All the patients included in our analysis completed the clinical follow-up.

The 6- and 12-months clinical data showed an mRS score 0–1 in 96,7% of the patients with the cumulative morbidity-mortality being 3,3%. One patient in the HSC group died of complications related to SAH and one of the BPC group had an mRS score of 2. In the remaining patients no changes were found in the mRS at the 12-months clinical follow-up.

According to these data, there were no statistically significant differences between the two types of coils (BPC and HSC group) of the mRS values recorded during the baseline, the 6 and the12 months follow-ups.

## Discussion

Consistent with the results of a current meta-analysis,^6^ our study provides evidence that HSC are more efficient in treating large and giant aneurysm with better mid-term outcomes when compared with BPC. Our overall treatment results are also matching with published data of four randomized controlled trials^7–10^ and three registries^11–13.^ The reported procedural complication rates in these studies ranged from 4.7 to 22% (vs. 9.8% in our study). The early mortality rates varied between 0 and 2.2% (none in our cohort). Residual aneurysms at the end of the procedure were observed between 5.4 and 37.3% of the cases part of these studies (none in our study). In our study the use of HSC was not associated with an increased incidence of parent vessel perforation, parent vessel occlusion, procedural aneurysm rupture, and thromboembolic events. Further on, recurrence was a high-profile and controversial issue among different clinical trials and systematic reviews: HELPS (Hydrocoil Endovascular aneurysm occlusion and Packing Study),^14–16^ GREAT (German--French Randomized Endovascular Aneurysm Trial)^8,17^ and Serafin's systematic review^18^ illustrated that hydrogel coils resulted in a lower rate of recurrence when compared to BPC. In contrast, no significant differences between coil types were observed through Poncyljusz's RCT^19^ and PRET (Patients prone to Recurrence after Endovascular Treatment).^20–22^ In our study 2^nd^ generation HSC significantly reduced the recurrence of large aneurysms without an increased intra- and periprocedural risk when compared to BPC.

The main limitations of this study were the retrospective fashion, a relatively small sample size and the need of adjunctive stents for some large-necked aneurysm, with the risk of modifying the effect of aneurysm sac occlusion and recurrence rates. However, the number of cases in which a stent was implanted was well balanced between the two arms of the study.

## Conclusions

By comparing the 2^nd^ generation HSC with BPC in the endovascular treatment of large brain aneurysms in our single-centre experience, we conclude that they are safe and effective by improving complete occlusion rates and reducing residual aneurysm neck rates at mid-term follow-up. In addition, they showed a lower rate of recurrence when compared to BPC and therefore could positively affect clinical outcome.

The Authors declare that there is no conflict of interest This research received no specific grant from any funding agency in the public, commercial, or not-for-profit sectors.
